# Benincaside A Induces p53-Dependent Transactivation and Fas/CD95-Mediated Apoptosis in HCT 116 Human Colorectal Cancer Cells

**DOI:** 10.3390/cimb48060635

**Published:** 2026-06-18

**Authors:** Jai-Sing Yang, Kun-Ching Cheng, Yu-Hsiu Chuang, Ping-Chung Kuo, Tian-Shung Wu

**Affiliations:** 1 Department of Medical Research, China Medical University Hospital, China Medical University, Taichung 404, Taiwan; jaisingyang@gmail.com; 2 Taiwan Sugar Research Institute, Tainan 701, Taiwan; lando.cheng@gmail.com; 3 Department of Chemistry, National Cheng Kung University, Tainan 701, Taiwan; melody19860212@gmail.com; 4 School of Pharmacy, College of Medicine, National Cheng Kung University, Tainan 701, Taiwan

**Keywords:** benincaside A (BA), HCT116 human colorectal cancer cell, apoptosis, reactive oxygen species (ROS)

## Abstract

An undescribed seco-kaurane diterpenoid, benincaside A (BA), was isolated from the seeds of *Benincasa hispida*. The seeds of *B. hispida* have been traditionally used in folk medicine and previous studies have reported anti-tumor potential in *B. hispida* seed extracts. Accordingly, we investigated the cytotoxicity and underlying mechanisms of BA in colorectal cancer cells. BA inhibited growth in HT29, Colo205, HCT116, and CT26 colorectal cancer cells, as determined by 3-(4,5-Dimethylthiazol-2-yl)-2,5-diphenyltetrazolium bromide (MTT) assay, while showing no toxicity toward normal human umbilical vein endothelial cells (HUVEC) and human fibroblast WS-1 cells. In HCT116 cells, BA-induced deoxyribonucleic acid (DNA) damage and apoptosis, as evidenced by morphological changes, 4,6-diamidino-2-phenylindole dihydrochloride (DAPI) staining, and assays of caspase-8 and caspase-3 activities. BA triggered apoptotic cell death via the extrinsic pathway, as indicated by elevated caspase-8 and caspase-3 activities. Intracellular reactive oxygen species (ROS) generation was observed in BA-treated HCT116 cells. The growth-inhibitory effects were significantly attenuated by pretreatment with *N*-acetylcysteine (NAC, an antioxidant), caffeine (an ATM kinase inhibitor), z-VAD-fmk (pan-caspase inhibitor), or z-IETD-fmk (caspase-8-specific inhibitor). Colorimetric assays confirmed increased caspase-8 and caspase-3 activities in BA-treated cells. This study is the first to report ROS-dependent signaling as a key mechanism underlying BA-induced cell death in HCT116 human colorectal cancer cells.

## 1. Introduction

Apoptosis, programmed cell death, is a biological process that plays an important role in the development and tissue homeostasis. Numerous studies have shown that the intrinsic and extrinsic pathways are involved in apoptotic cell death [1]. The extrinsic pathway (also called the death receptor pathway) is triggered by the binding of extrinsic ligands to surface receptors, including CD95/Fas, tumor necrosis factor (TNF), and death receptors (DRs), leading to the activation of caspase-8, which in turn activates caspase-3 [2]. The intrinsic pathway (also called the mitochondrial pathway) is triggered by various stimuli that cause cellular damage, leading to decreased mitochondrial membrane potential and the activation of caspase-9, which in turn activates caspase-3 [3]. The p53 tumor suppressor protein performs an important role in inducing apoptosis. The p53-inducible pro-apoptotic genes trigger apoptosis through both extrinsic and intrinsic pathways. Many proapoptotic proteins, including Fas/CD95 [4], death receptors (DRs) [5], PUMA, Bax, Bik, and Bid [6], have been reported to be transcriptional targets of p53.

In traditional folk medicine, the seeds of *B. hispida* have been used to promote blood circulation, as an anti-tussive herb, and as a health food. In previous reports, the extract of *B. hispida* seeds exhibited inhibitory effects on histamine secretion, anti-angiogenic and anti-tumor activity, and enhanced immune responses [7,8]. In this study, we characterized an undescribed metal-containing seco-kaurane diterpenoid, benincaside A (BA), from the seeds of *B. hispida* and investigated the molecular mechanism of apoptosis induction by BA in HCT116 cells. The results suggested that BA targeted tumor microvasculature via p53-mediated upregulation of Fas/CD95 and should be a potential lead candidate for further drug discovery.

## 2. Materials and Methods

### 2.1. Chemicals and Reagents

Agarose, caffeine, DAPI (4,6-diamidino-2-phenylindole dihydrochloride), dimethyl sulfoxide (DMSO), MTT (3-(4,5-Dimethylthiazol-2-yl)-2,5-diphenyltetrazolium bromide), *N*-acetylcysteine (NAC), potassium phosphate, Triton X-100, and ribonuclease-A were obtained from Sigma-Aldrich Corp. (St. Louis, MO, USA). H_2_DCF-DA was obtained from Invitrogen Life Technologies (Carlsbad, CA, USA). RPMI-1640 medium, McCoy’s Sa medium, L-glutamine, fetal bovine serum (FBS), trypsin-EDTA, penicillin, and streptomycin were obtained from Invitrogen. Caspase-3, -8, and -9 activity assay kits, caspase-3 specific inhibitor (Z-DEVD-FMK), caspase-8 specific inhibitor (Z-IED-FMK), and caspase-9 specific inhibitor (Z-LEHD-FMK) were purchased from R&D Systems (Minneapolis, MN, USA). Electrophoresis materials and chemicals were obtained from Bio-Rad (Hercules, CA, USA). The p53 small interfering RNA (siRNA) and Lipofectamine 2000 were obtained from Invitrogen Life Technologies.

### 2.2. Extraction and Isolation

The seeds of *B. hispida* were purchased in 2009 from Chuang Song Zong Pharmaceutical Co., Ltd., Pingtung, Taiwan, and the plant material was identified and authenticated by Prof. C. S. Kuoh, Department of Bioscience, National Cheng Kung University, Tainan, Taiwan. A voucher specimen (TSWu 200900701) has been deposited in the herbarium of the School of Pharmacy, College of Medicine, National Cheng Kung University, Tainan, Taiwan. The seeds of *B. hispida* (20 kg) were pulverized and extracted with hot water under reflux. The filtrate was concentrated under reduced pressure to obtain the crude extract solution. Further partition of this solution between CHCl_3_ and water afforded the CHCl_3_ (300 g) and water layers (600 g), respectively. The water layer was chromatographed on a Diaion HP-20 column eluted with H_2_O, followed by step gradients with MeOH to obtain seven fractions (Fr. 1–7). Fr. 5 was subjected to Diaion HP-20 column using a stepwise gradient of H_2_O-MeOH to afford six subfractions (Fr. 5.1–5.6). Subfraction 5.4 was further purified by silica gel using EtOAc-MeOH-H_2_O (5:1:0.1) to provide BA (158.5 mg).

#### Spectral Data of Benincaside A

**Figure 1:**
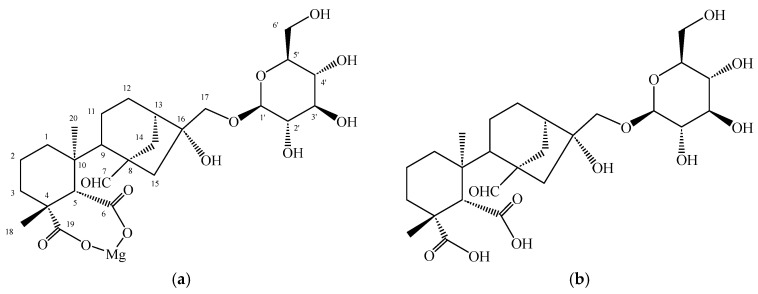
The chemical structures of (**a**) benincaside A, and (**b**) its acid-hydrolyzed product.

The structure of benincaside A is shown in Figure 1, and the spectral data are as follows.

Colorless powder; [α]_D_^25^ + 75 (*c* 0.1, MeOH); UV (MeOH) λ_max_ (log ε): 272 (3.12), 228 (2.79) nm; IR (neat) *ν*_max_ 3387, 2959, 1709, 1636, 1585, 1481, 1439 cm^−1^; ^1^H-NMR (CD_3_OD, 400 MHz) *δ* 9.64 (1H, s, CHO), 4.32 (1H, d, *J* = 7.6 Hz, H-1′), 4.18 (1H, d, *J* = 10.0 Hz, H-17a), 3.86 (1H, d, *J* = 10.8 Hz, H-6′a), 3.70 (1H, d, *J* = 10.8, 4.4 Hz, H-6′b), 3.58 (1H, d, *J* = 10.0 Hz, H-17b), 2.63 (1H, s, H-5), 1.20 (3H, s, H-18), 0.98 (3H, s, H-20), 0.89 (1H, m, H-3b); ^13^C-NMR (CD_3_OD, 100 MHz) *δ* 207.1 (C-7), 183.0 (C-19), 180.8 (C-6), 104.8 (C-1′), 80.9 (C-16), 77.8 (C-5′), 77.6 (C-3′), 75.0 (C-2′), 74.1 (C-17), 71.4 (C-4′), 64.6 (C-5), 62.5 (C-6′), 60.3 (C-8), 47.9 (C-9), 46.3 (C-13), 45.8 (C-4), 43.9 (C-10), 38.9 (C-3), 37.2 (C-1), 30.7 (C-14), 29.5 (C-18), 26.9 (C-2), 23.1 (C-20), 20.6 (C-15); ESI-MS *m*/*z* 589 [M + Na]^+^, 567 [M + H]^+^; HRESIMS for the acid hydrolyzed product, *m*/*z* 545.2593 ([M + H]^+^ calcd for C_26_H_40_O_12_, 545.2598).

### 2.3. Cell Culture

Human colorectal cancer cell lines HT29 (BCRC Number: 60157), Colo205 (BCRC Number: 60054), HCT116 (BCRC Number: 60349), murine colorectal cancer cell line CT26 (BCRC Number: 60443), normal human umbilical vein endothelial cells (HUVEC) (BCRC Number: H-UV001), and normal human fibroblast WS-1 cells (BCRC Number: 60300) were obtained from the Food Industry Research and Development Institute (BCRC, Hsinchu, Taiwan). HCT116 cells were cultured in T75 flasks with McCoy’s Sa medium. HT29, Colo205, and CT26 cells were cultured in RPMI-1640 medium. HUVEC and WS-1 cells were cultured in IMDM medium. All cell culture media contained 2 mM L-glutamine and were supplemented with 10% FBS, 100 Units/mL penicillin, and 100 μg/mL streptomycin and the cells were maintained at 37 °C under a humidified 5% CO_2_ atmosphere.

### 2.4. Cell Viability and Morphological Changes

Cells were placed in 96-well cell culture plates with an initial concentration of 2 × 10^4^ cells/well and exposed to BA at various concentrations (10, 20, 30, 40, and 50 μM) or 0.1% DMSO as a vehicle control for 48 h. After a 48 h incubation period, a volume of 100 μL MTT (0.5 mg/mL) was added to the wells for 4 h. The growth medium was removed, and the formazan crystals formed were dissolved with isopropanol/HCl and measured at 490 nm wavelength by ELISA reader [9,10]. For morphological changes, HCT 116 cells were cultured in 12-well plates at 2 × 10^5^ cells/well and treated with or without 30 μM of BA for 48 h. A phase-contrast microscope directly examined morphological changes in BA-treated cells [9,10]. All results were performed in triplicate independent experiments.

### 2.5. DAPI Staining

HCT 116 cells were cultured in 12-well plates at a density of 2 × 10^5^ cells/well and incubated with or without 30 μM of BA for 48 h. Cells were harvested by centrifugation, stained by 4′,6-diamidino-2-phenylindole dihydrochloride (DAPI), and assessed by fluorescence microscopy [11,12].

### 2.6. Production of Reactive Oxygen Species (ROS)

HCT 116 cells in 12-well plates at a density of 2 × 10^5^ cells/well were exposed to 30 μM of BA for 0, 2, 4, 8, and 12 h. At the end of the experiment, cells were harvested and stained with 5 μM 2,7-dichlorodihydrofluorescein diacetate (H2DCF-DA, a ROS indicator as a fluorescent probe) at 37 °C for 30 min. ROS was analyzed for fluorescence intensity by using flow cytometry. The median fluorescence intensity (MF) was quantified using BD CellQuest Pro software (version 4.0.2; Becton, Dickinson) as previously described [12,13]. All results were obtained from independent triplicate experiments.

### 2.7. Assays for Caspase-3, Caspase-8, and Caspase-9 Activities

Caspase-3, -8, and -9 activities were assessed according to the manufacturer’s instructions for caspase colorimetric kits (R&D Systems Inc., Minneapolis, MN, USA). HCT 116 cells were cultured in 75T flasks at a density of 5 × 10^6^ cells and incubated with 30 μM of BA for 0, 12, 24, 36, and 48 h. Cells were harvested in 50 μL of lysis buffer. After centrifugation, the supernatant containing 100 μg protein was incubated with caspase-3 substrate (Ac-DEVD-pNA), caspase-8 substrate (Ac-IETD-pNA), and caspase-9 substrate (Ac-LEHD-pNA) in reaction buffer. All samples were then incubated at 37 °C for 1 h. Levels of released pNA were measured with an Anthos 2001 ELISA reader (Biochrom, Cambridge, UK) as previously described [14,15]. All results were obtained from independent triplicate experiments.

### 2.8. Statistical Analysis

All data were expressed as mean ± SEM from at least three separate experiments. Statistical calculations of the data were obtained using one-way ANOVA followed by Tukey’s test. Statistical significance was set at *p* < 0.001 (***) and was taken as significant.

## 3. Results

### 3.1. Characterization of Benincaside A (BA)

BA (Figure 1a) was characterized from *B. hispida* seeds, belonging to the family Cucurbitaceae. BA was purified as an amorphous powder with a positive optical rotation ([α]_D_^25^ + 75). Although the molecular adduct ion was observed in electrospray-mass spectrometry (ESI-MS) analysis (*m*/*z* 567 for [M + H]^+^) (Figure S1), high resolution mass spectrometry (HRMS) analysis did not reveal any significant signals. Therefore, the sample was acid hydrolyzed and chromatographed through a Sephadex LH-20 column. The water eluent from the acid-hydrolyzed product was analyzed by atomic absorption spectroscopy (AAS), which indicated the presence of magnesium ions (Figure S2). The lipophilic eluent showed a protonated molecular ion at *m*/*z* 545.2593 (Figure S3), suggesting a molecular formula of C_26_H_40_O_12_ (calcd for [M + H]^+^, *m*/*z* 545.2598; Figure 1b). In summary, the complete formula of BA could be proposed as C_26_H_38_MgO_12_. The UV absorption maxima at 272 and 228 nm suggested the presence of carbonyl groups (Figure S4) [16]. The IR absorption bands at 1709 and 1636 cm^−1^ were attributed to the carbonyl and carbon-carbon double bond functionalities (Figure S5). In its ^1^H NMR spectrum (Figure S6), two singlet methyls at δ_H_ 1.20 (CH_3_-18) and 0.98 (CH_3_-20) indicated the diterpenoid basic skeleton, and it was further suggested as a kaurane diterpenoid glycoside by considering the molecular formula and all the ^1^H and ^13^C NMR signals (Figures S6 and S7). One methyl group was oxidized to a carboxylic acid, as evidenced by a ^13^C NMR signal at δ_C_ 183.0 (C-19), with an HMBC correlation between CH_3_-20 and C-19. The presence of a typical C-17 hydroxymethyl group was revealed by two mutually coupling doublets at δ_H_ 4.18 (H-17a) and 3.58 (H-17b). This group was connected to a glucose moiety (δ_C_ 104.8, 77.8, 77.6, 75.0, 71.4, and 62.5), as evidenced by the downfield shift in C-17 (δ_C_ 74.1) and HMBC correlation between H-17 and C-1′ (δ_C_ 104.8). The unique structure characteristic of this compound was the cleavage of the B-ring, which was supported by the presence of an aldehyde signal at δ_H_ 9.64 (s, C-7) and one carboxylic acid group at δ_C_ 180.8 (C-6), and the disappearance of CH_2_-6 and -7. This was also confirmed by 2D NMR analysis, which showed ^2^J, ^3^J-HMBC correlations from H-5 (δ_H_ 2.63, s) to C-6, and from CH_2_-15 to C-7, respectively. All these experimental data revealed that the C-6/C-7 bond in the kaurane diterpene experienced oxidative cleavage. Accordingly, the structure of BA was assigned as shown (Figure 1a).

### 3.2. BA Induced Growth Inhibition in Colorectal Cancer Cells

**Figure 2:**
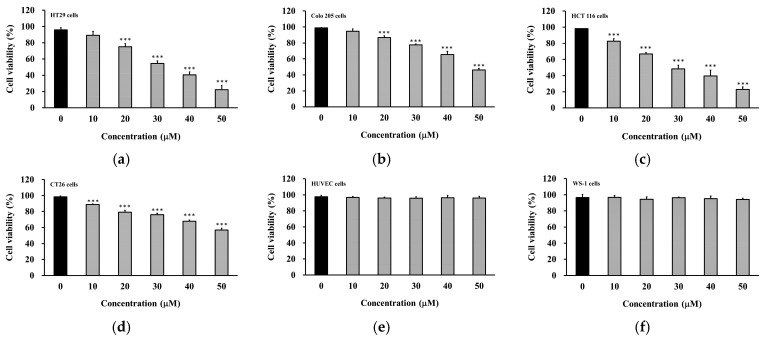
Effects of BA on cell viability in human colorectal cancer cell lines and normal cells. Cells, including (**a**) HT29, (**b**) Colo 205, (**c**) HCT 116, and (**d**) CT26 colorectal cancer cells, as well as (**e**) normal human umbilical vein endothelial cells (HUVEC) and (**f**) normal human fibroblast WS-1 cells, were treated with 0, 10, 20, 30, 40, or 50 μM BA for 48 h. Cell viability was then assessed by MTT assay as described in Materials and Methods. The values presented are the mean ± SD (*n* = 3) from three independent experiments. *** *p* < 0.001 was significantly different from vehicle control cells.

The growth inhibition effect of BA on colorectal cancer cells was investigated in this study. As shown in Figure 2, BA inhibited cell growth dose-dependently in human colorectal cancer cell lines HT 29 (Figure 2a), Colo 205 (Figure 2b), HCT 116 (Figure 2c), and murine colorectal cancer cell line CT 26 (Figure 2d). It was non-toxic for normal human umbilical vein endothelial cells (HUVEC) (Figure 2e) and normal human fibroblast WS-1 cells (Figure 2f). The half-maximal inhibitory concentrations (IC_50_) for 48 h treatment of BA in HT 29, Colo 205, HCT 116, and CT 26 cell lines were 37.65 ± 2.87, 48.77 ± 1.99, 30.18 ± 2.07, and 54.89 ± 2.76 μM, respectively. Based on the above results, a BA concentration of 30 μM was used in all subsequent experiments in the present study.

### 3.3. BA Triggered DNA Damage and Apoptosis in HCT 116 Cells

**Figure 3:**
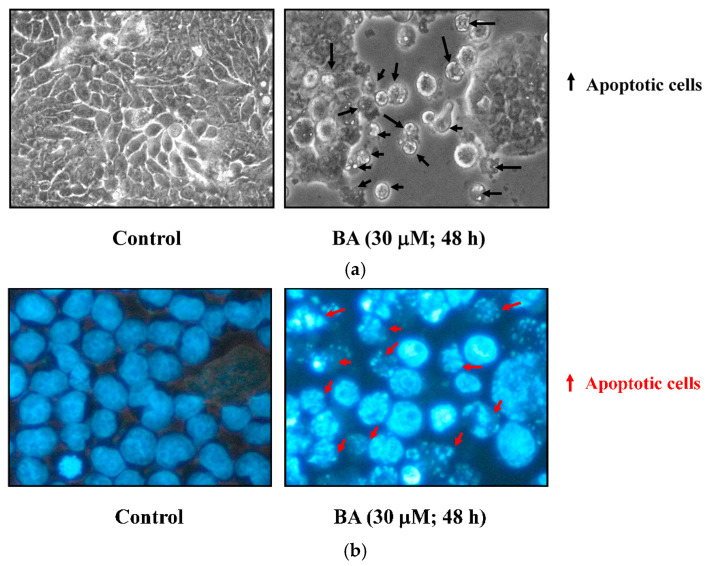
Effects of BA on morphological changes and DNA condensation in HCT 116 cells. (**a**) Following treatment with 30 μM BA for 48 h, morphological changes were examined and photographed under phase-contrast microscopy. (**b**) DNA condensation was assessed by DAPI staining, as described in Materials and Methods.

BA-induced reduction in cell viability might be fully mediated by DNA damage and apoptosis. As shown in Figure 3a, the concentration of 30 μM of BA increased cell rounding and morphological changes in HCT 116 cells after a 48 h treatment. Figure 3b showed that DAPI staining induced nuclear condensation and apoptotic bodies in HCT 116 cells. BA induced apoptosis because of the occurrence of a DNA ladder in HCT 116 cells. The results suggested that BA induced cell death via apoptosis and DNA damage in HCT 116 cells.

### 3.4. BA Promoted ROS Production in HCT 116 Cells

**Figure 4:**
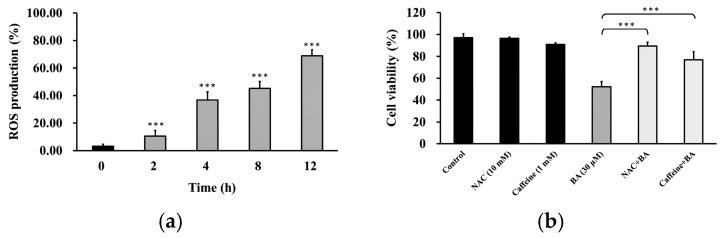
Effects of BA on reactive oxygen species (ROS) levels in HCT 116 cells. (**a**) ROS levels in HCT 116 cells treated with 30 μM BA were measured by flow cytometry at 0, 2, 4, 8, and 12 h and are shown as percentages relative to the untreated control at 0 h, which was set to 100%. *** *p* < 0.001 versus vehicle control (0 h) cells. (**b**) Pretreatment with NAC or caffeine restored cell viability in HCT 116 cells treated with 30 μM BA, as assessed by MTT assay described in Materials and Methods. *** *p* < 0.001 versus BA-treated cells.

**Figure 5:**
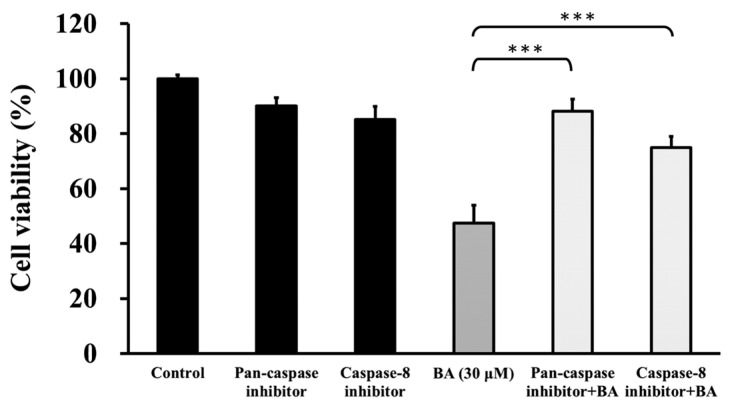
Effects of BA on caspase activity in HCT 116 cells. Cells were pretreated with a caspase-8 inhibitor or a pan-caspase inhibitor for 1 h, followed by exposure to 30 μM BA for 48 h. Cell viability was assessed by MTT assay as described in Materials and Methods, and the results are expressed as percentages relative to the untreated control (100%). The values presented are the mean ± SD (*n* = 3) from three independent experiments. *** *p* < 0.001, significantly different from the 30 μM BA treated group.

To examine the effects of BA on ROS production of HCT 116 cells, specific ROS fluorescence probes (H_2_DCF-DA) were used to detect the level of ROS. As shown in Figure 4a, BA increased intracellular ROS levels noticeably. Cells were pretreated with *N*-acetylcysteine (NAC, an antioxidant) and caffeine (an ATM kinase inhibitor), which significantly reduced BA-induced cell death and growth effects (Figure 4b). In 2011, Mellert et al. reported that p53 was an important regulator of the apoptotic response to oxidative DNA damage [17]. To confirm the involvement of the extrinsic apoptotic pathway possibly involved in BA-induced apoptosis in HCT 116 cells, specific inhibitors of caspase-8 (z-IETD-fmk) and pan-caspase (z-VAD-fmk) significantly prevented the effects of BA-induced growth inhibition when pre-treated (Figure 5). The results indicate that the caspase-8-dependent death receptor pathway may play a key role in BA-induced apoptotic death in HCT 116 cells.

### 3.5. BA Stimulated Caspase-8 and Caspase-3 Activities in HCT116 Cells

**Figure 6:**
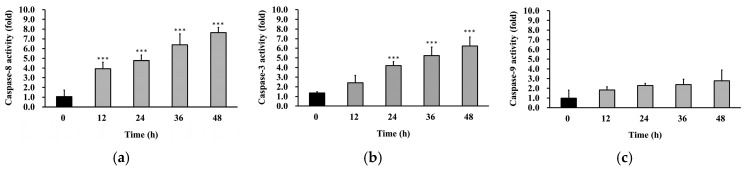
Effects of BA on caspase-8, -9, and -3 activities in HCT 116 cells. Cells were treated with 30 μM BA for 0, 12, 24, 36, or 48 h, after which (**a**) caspase-8, (**b**) caspase-9, and (**c**) caspase-3 activities were measured by caspase activity assay, as described in Materials and Methods. *** *p* < 0.001 was significantly different from vehicle control (0 h) treated cells.

The extrinsic apoptotic pathway may be involved in BA-induced apoptosis in HCT 116 cells; thus, we investigated the activities of caspases 8, 9, and 3. As shown in Figure 6, the concentration of 30 μM BA caused an increase in caspase-8 (Figure 6a) and caspase-3 (Figure 6c) activities after 0, 12, 24, 36, and 48 h treatment, and both caspase activities were significantly increased in a time-dependent manner. Caspase-9 activity was not significantly different from that of untreated cells (Figure 6b). Based on the above data, the activation of caspase-8 and caspase-3 may be involved in BA-induced death receptor-mediated apoptosis in HCT 116 cells.

## 4. Discussion

*B. hispida* is a very popular vegetable in Taiwan. Many studies have been conducted to reveal its anti-inflammatory, diuretic, anti-angiogenic, and anti-cancer activities. It has been demonstrated that the major constituents of *B. hispida* are carotenes, flavonoids, glycosides, saccharides, triterpenoids, uric acids, vitamins, and β-sitosterol. In this study, benincaside A (BA) (Figure 1), a previously undescribed compound, was isolated from *B. hispida*. However, no information is available to address BA-induced growth inhibition and cell death in human cancer cells. Therefore, this new investigation will help further elucidate the undiscovered biological properties of this novel anti-tumor BA in human colorectal cancer cells. In this study, we investigated the molecular targets and mechanisms underlying BA-induced apoptosis in HCT 116 cells. Also, the anticancer activity of BA is not strong, but it is less cytotoxic to normal cells (HUVEC and WS-1) (Figure 2e,f). We further examined the effects of BA in the normal colon epithelial cell line CCD 841 CoN (ATCC CRL-1790), and found that BA showed minimal effects on cell viability (Figure S8), morphological changes (Figure S9), ROS levels (Figure S10), and caspase-3 activity (Figure S11), indicating selective anticancer activity in HT116 cells. Our results suggest that BA-induced apoptotic cell death in HCT 116 cells occurs through ROS-p53-dependent transactivation and the extrinsic apoptotic pathway.

Activation of the extrinsic initiator caspase-8 triggers downstream caspase-3, which induces cell apoptosis [18,19,20]. Figure 6 showed that the activities of caspase-8 and -3 were increased in BA-treated HCT 116 cells in a time-dependent manner. Pre-incubation with specific pan-caspase and caspase-8 inhibitors increased cell viability (Figure 5). Thus, caspase-8 played a key role in BA-induced apoptosis. These accumulating data suggested BA-triggered apoptosis in HCT116 cells through death receptor-dependent signaling pathways.

Recently, several reports indicated that ATM/p53 is a key enzyme in maintaining genome integrity by coordinating cell cycle arrest, DNA damage response, DNA repair, and apoptosis [21,22,23]. DNA damage stress triggered activation of the ataxia telangiectasia mutated (ATM) signaling pathways, including ATM autophosphorylation and phosphorylation of ATM target substrates, p53, H_2_AX, and Chk2 [23,24]. Our results confirmed previous reports [25] and showed that BA increased ROS levels in HCT116 cells (Figure 4a). BA-induced DNA damage by DAPI staining (Figure 3). Pretreatment with NAC or caffeine in BA-treated HCT 116 cells restored cell viability (Figure 4b), suggesting the functional involvement in ROS and DNA damage. The ATM/p53 activation induced pro-apoptotic genes transcription such as Fas/CD95 receptor [5,23].

While our current study establishes a consistent profile of apoptosis and genotoxic stress through morphological observation, DAPI staining, Caspase-8 activity assays, and Comet assay analysis, we acknowledge several limitations. First, although Caspase-8 activation is a hallmark of the extrinsic apoptotic pathway, further investigations—such as co-immunoprecipitation for DISC complex analysis or assessment of membrane receptor expression—are required to definitively confirm the specific involvement of Fas/CD95 or other death receptors. Furthermore, quantitative approaches, such as Annexin V/PI flow cytometry, would provide more robust verification of our findings. Finally, we recognize that our findings are preliminary and that the absence of in vivo validation represents a significant limitation. Future studies employing animal models will be essential to evaluate the pharmacokinetics, bioavailability, and therapeutic efficacy of BA within a more complex biological environment.

## 5. Conclusions

In conclusion, BA triggered ATM/p53-dependent DNA damage through the extrinsic apoptotic pathway. The present studies demonstrate the important molecular mechanisms underlying anticancer activity, and BA may be a potential lead compound worthy of further investigation in the treatment of human colorectal cancer.

## Data Availability

The raw data supporting the conclusions of this article will be made available by the authors on request.

## Supplementary Materials

The following supporting information can be downloaded at: https://www.mdpi.com/article/10.3390/cimb48060635/s1.

## Abbreviations

The following abbreviations are used in this manuscript:

BA	benincaside A
DAPI	4,6-diamidino-2-phenylindole dihydrochloride
DCFH-DA	2′,7′-dichlorofluorescin diacetate
DiOC6	3,3′-dihexyloxacarbocyanine iodide
DMSO	dimethyl sulfoxide
PI	propidium iodide
ΔΨm	mitochondrial membrane potential
