# Supplementary material for: Benincaside A Induces p53-Dependent Transactivation and Fas/CD95-Mediated Apoptosis in HCT 116 Human Colorectal Cancer Cells
Source: Curr Issues Mol Biol. 2026 Jun 18;48(6):635. doi: 10.3390/cimb48060635 (PMC13297861; doi:10.3390/cimb48060635)

Supplementary Materials

Benincaside A Induces p53-Dependent Transactivation and Fas/CD95-Mediated Apoptosis in HCT 116 Human Colorectal Cancer Cells

Jai-Sing Yang [1,†], Kun-Ching Cheng [2,†], Yu-Hsiu Chuang [3], Ping-Chung Kuo [4,*], and Tian-Shung Wu [4,*]

[1] Department of Medical Research, China Medical University Hospital, Taichung, 404, Taiwan; jaisingyang@gmail.com

[2] Taiwan Sugar Research Institute, Tainan 701, Taiwan; lando.cheng@gmail.com

[3] Department of Chemistry, National Cheng Kung University, Tainan 701, Taiwan; melody19860212@gmail.com

[4] School of Pharmacy, College of Medicine, National Cheng Kung University, Tainan 701, Taiwan

* Correspondence: z10502016@ncku.edu.tw; Tel.: 886-6-2353535 ext 6806 (P.-C.K.); tswu@mail.ncku.edu.tw; Tel.: 886-6-2757575 ext 65333 (T.-S.W.)

† These authors contributed equally to this work.

Contents

Figure S1. ESI-MS of benincaside A.

Figure S2. AAS examination data of acid hydrolyzed product of benincaside A.

Figure S3. HR-ESI-MS of acid hydrolyzed product of benincaside A.

Figure S4. UV spectrum of benincaside A.

Figure S5. IR spectrum of benincaside A.

Figure S6. ^{1}H-NMR of benincaside A.

Figure S7. ^{13}C-NMR of benincaside A.

Figure S8. Effects of BA on cell viability in normal colon epithelial CCD 841 CoN cells (ATCC CRL-1790).

Figure S9. Effects of BA on morphological changes in normal colon epithelial CCD 841 CoN cells.

Figure S10. Effects of BA on reactive oxygen species (ROS) levels in normal colon epithelial CCD 841 CoN cells.

Figure S11. Effects of BA on caspase-3 activity in normal colon epithelial CCD 841 CoN cells.

Figure S1. ESI-MS of benincaside A.

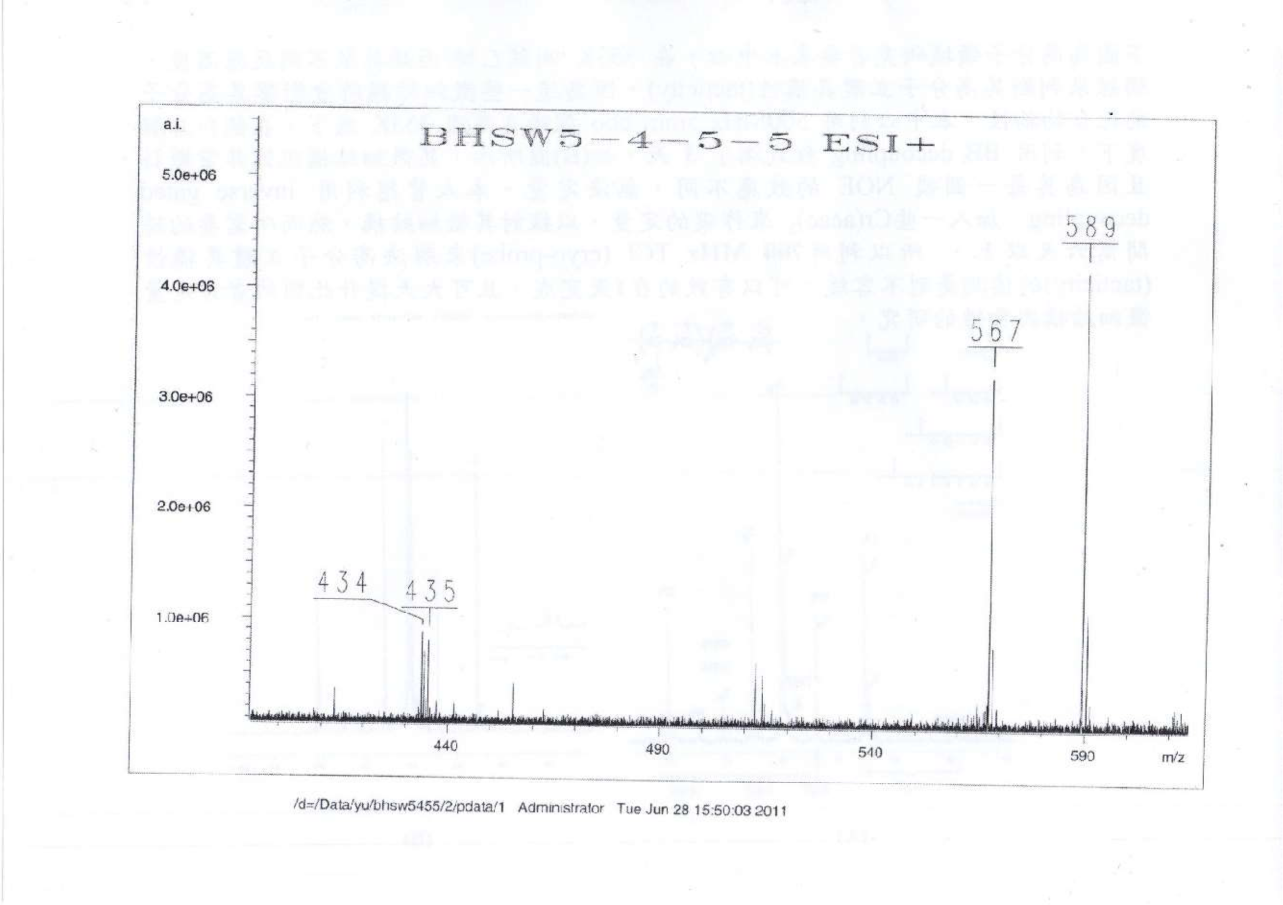

Figure S2. AAS examination data of acid hydrolyzed product of benincaside A.

Results (shown in ppm)			
Sample Code \ Element	Mg	Fe	Mn
BK	0.3150	0.388	0.0072
BHSW 5455 (test 1)	1.839	1.519	0.1258
BHSW 5455 (test 2)	1.524	1.131	0.1186

Figure S3. HR-ESI-MS of acid hydrolyzed product of benincaside A.

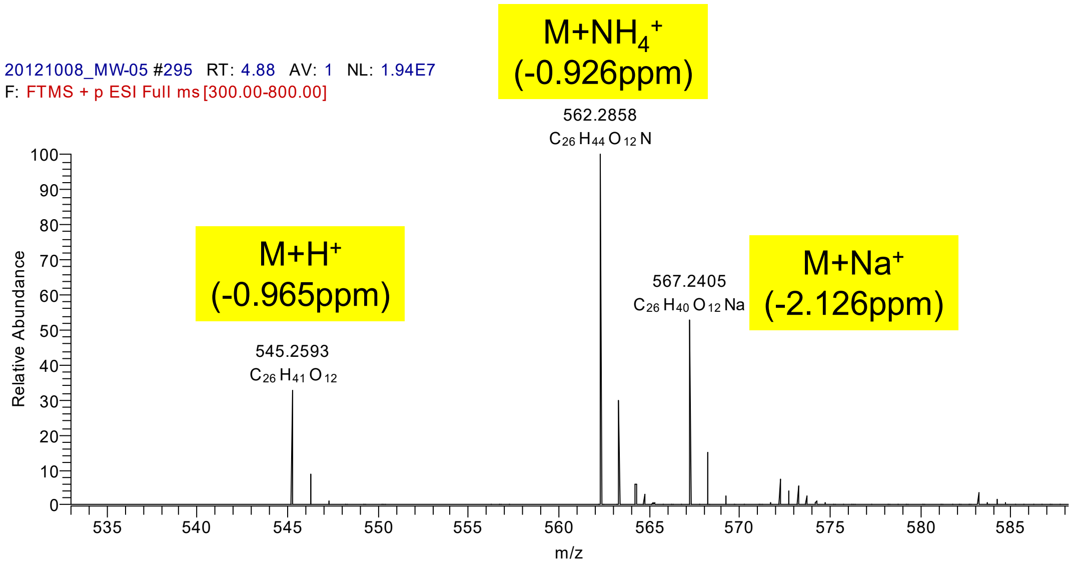

Figure S4. UV spectrum of benincaside A.

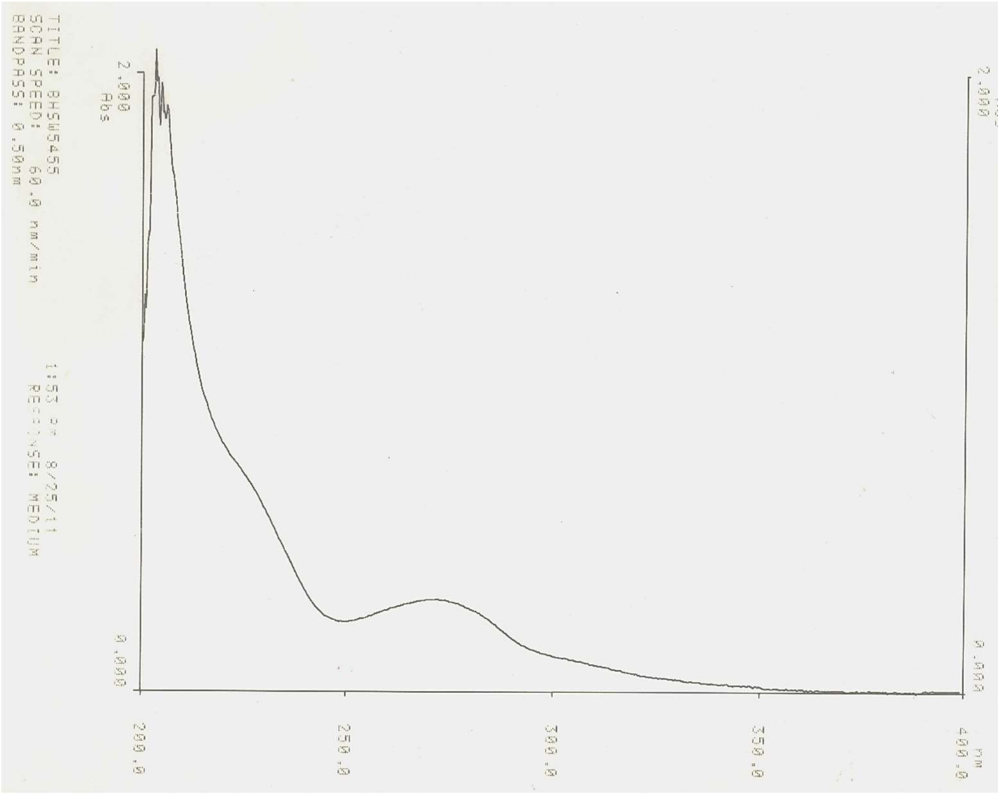

BHSW 5455

Molecular weight: 534 Sample weight: 1.3 mg

0.000487

λ_{max}	Abs	ε	log ε
228 nm	0.3010	618.208	2.79113
272 nm	0.6409	1316.31	3.11936

Figure S5. IR spectrum of benincaside A.

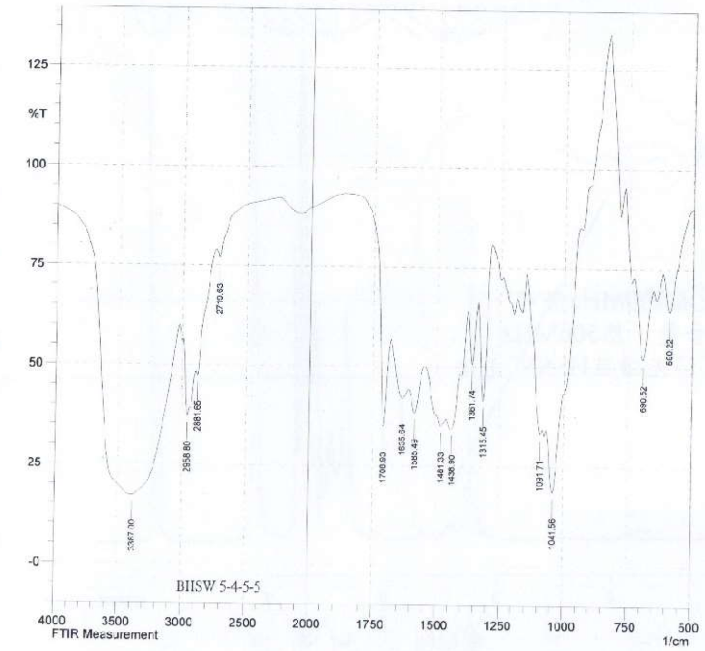

SHIMADZU

	Peak	Intensity	Corr. Inte	Base (H)	Base (L)	Area	Corr. Are
1	590.22	64.041	12.622	613.36	501.49	12.935	3.067
2	690.52	52.144	18.638	725.23	655.8	15.147	4.684
3	1041.56	18.62	26.742	1066.56	941.26	49.912	16.188
4	1091.71	33.274	5.794	1149.57	1083.99	20.097	0.55
5	1315.45	41.507	31.581	1338.6	1288.45	12.078	5.473
6	1361.74	50.709	14.219	1377.17	1342.46	8.435	1.966
7	1438.9	34.418	7.586	1462.04	1411.89	21.225	2.586
8	1481.33	35.093	4.078	1546.91	1465.9	32.337	2.285
9	1585.49	38.343	8.234	1608.63	1550.77	21.112	2.278
10	1635.64	41.867	6.557	1678.07	1612.49	22.439	2.389
11	1708.93	34.905	28.366	1832.38	1681.93	20.981	5.41
12	2719.63	75.793	3.026	2746.63	2256.71	26.081	0.437
13	2881.65	47.287	3.429	2893.22	2750.49	27.666	0.461
14	2958.8	37.023	7.359	2993.52	2943.37	18.451	1.728
15	3387	17.088	1.382	3398.57	3032.1	202.569	22.263

Comment;
FTIR Measurement

Date/Time; 2011/8/25 ,W,木 11:54:59
No. of Scans;
Resolution;
Apodization;
User; melody

Figure S6. ^{1}H-NMR of benincaside A.

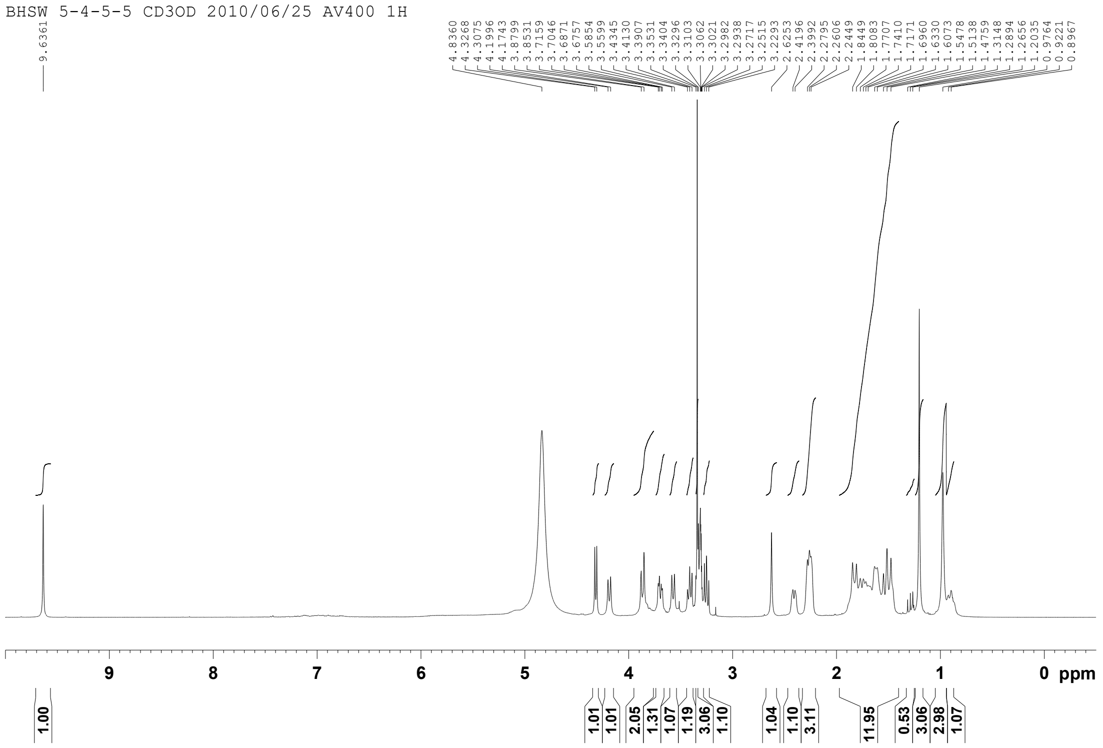

Figure S7. ^{13}C-NMR of benincaside A.**Figure S3.** UV spectrum of benincaside A.

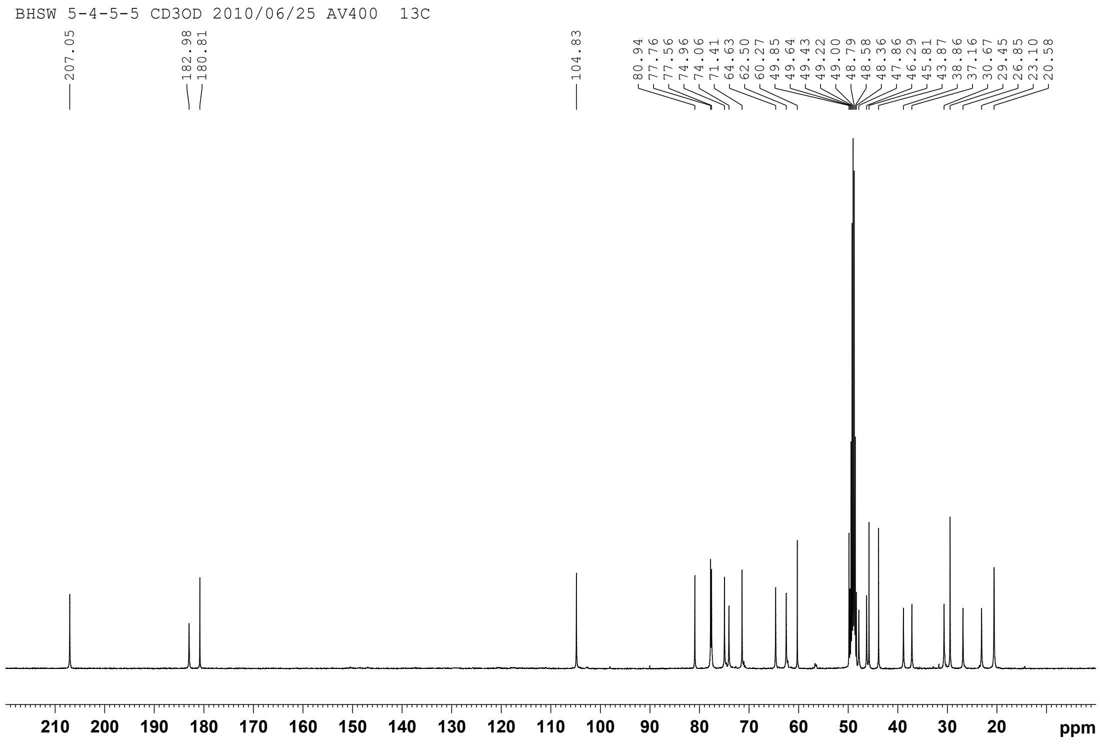

Figure S8. Effects of BA on cell viability in normal colon epithelial CCD 841 CoN cells (ATCC CRL-1790). Cell was treated with 0, 10, 20, 30, 40, or 50 μM BA for 48 h. Cell viability was then assessed by MTT assay as described in Materials and Methods.

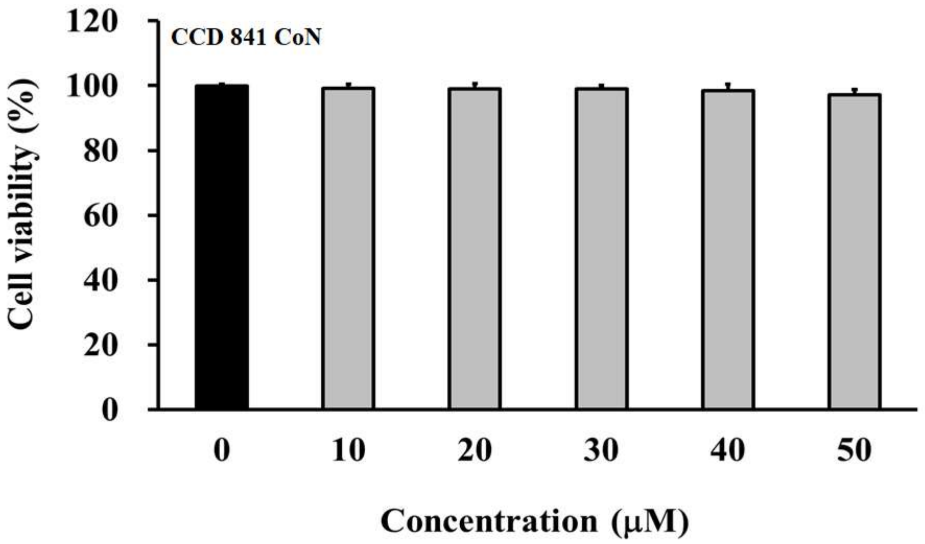

Figure S9. Effects of BA on morphological changes in normal colon epithelial CCD 841 CoN cells. Following treatment with 30 μM BA for 48 h, morphological changes were examined and photographed under phase-contrast microscopy.

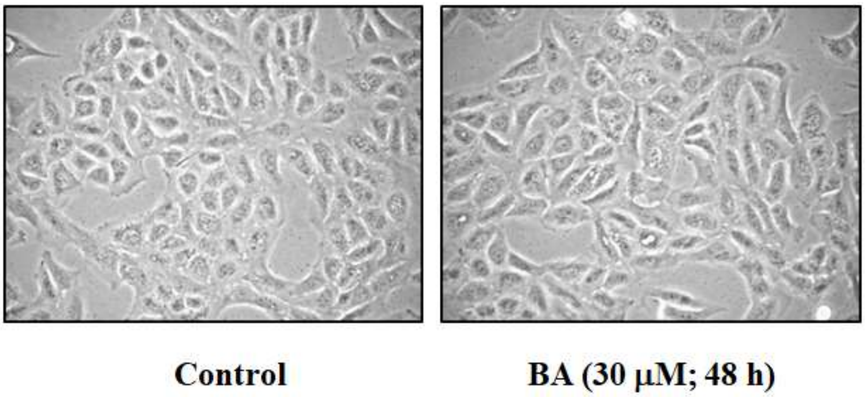

Figure S10. Effects of BA on reactive oxygen species (ROS) levels in normal colon epithelial CCD 841 CoN cells. ROS levels in CCD 841 CoN cells treated with 30 μM BA were measured by flow cytometry at 0, 2, 4, 8, and 12 h and are shown as percentages relative to the untreated control at 0 h, which was set to 100%.

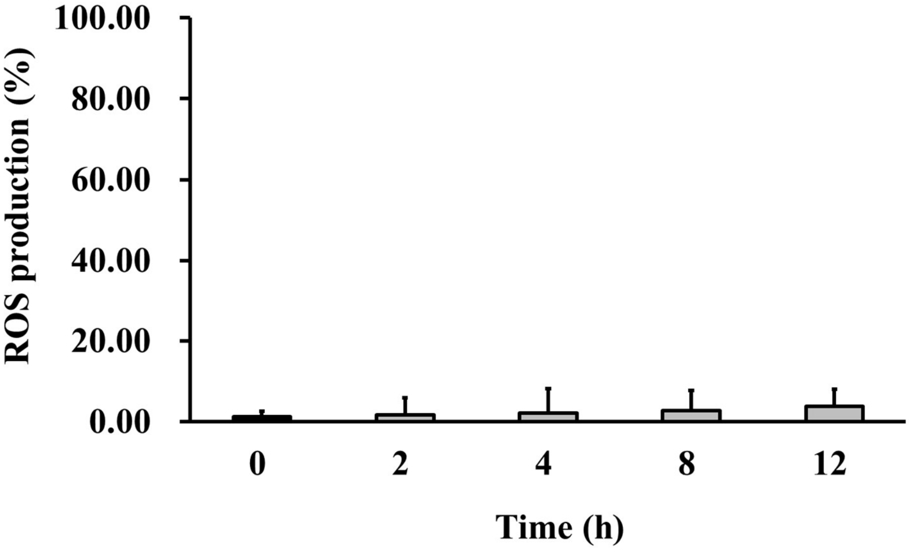

Figure S11. Effects of BA on caspase-3 activity in normal colon epithelial CCD 841 CoN cells. Cells were treated with 30 μM BA for 0, 12, 24, 36, or 48 h, after which caspase-3 activities was measured by caspase activity assay.

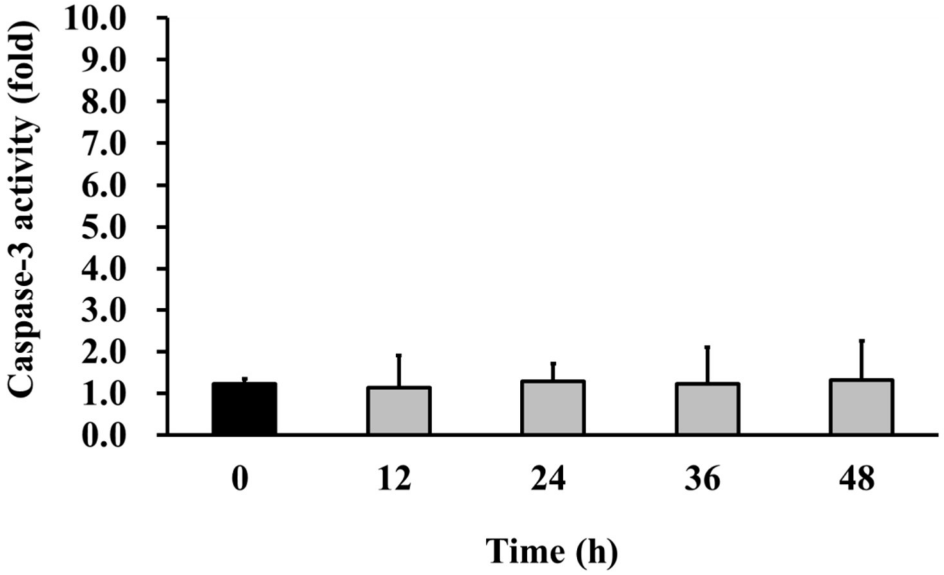

Supplement: Supplementary file 1 [file cimb-48-00635-s001.zip › cimb-4340937-supplementary.pdf]
